# Selection of autophagy or apoptosis in cells exposed to ER-stress depends on ATF4 expression pattern with or without CHOP expression

**DOI:** 10.1242/bio.20135033

**Published:** 2013-08-27

**Authors:** Hiroki Matsumoto, Shuichi Miyazaki, Satoshi Matsuyama, Masayuki Takeda, Makoto Kawano, Hiroshi Nakagawa, Kazuhiko Nishimura, Saburo Matsuo

**Affiliations:** 1 Laboratory of Toxicology, Course of Veterinary Science, Graduate School of Life and Environmental Biosciences, Osaka Prefecture University, 1-58, Rinku-Ourai-Kita, Izumisano 598-8531, Japan; 2 The Center for Advanced Research, Graduate School of Medical Science, Toho University of Medicine, Ota-ku, Omori-Nishi, 5-21-16, Tokyo 143-8540, Japan; 3 Laboratory of Veterinary Radiology, Course of Veterinary Science, Graduate School of Life and Environmental Biosciences, Osaka Prefecture University, 1-58, Rinku-Ourai-Kita, Izumisano 598-8531, Japan

**Keywords:** ER-stress, Autophagy, ATF4, CHOP, PERK pathway

## Abstract

Cells exposed to ER-stress undergo the Unfolded Protein Response (UPR) to avoid apoptosis, but may also activate autophagy. However, the signal for selection of one of these two protective responses is unknown. To clarify the key switch between autophagy and apoptosis, we examined the correlation of UPR-related signals with autophagy and/or apoptosis inductions in HepG2 cells exposed to three ER-stress inducers (NaF, tunicamycin, and thapsigargin) with time, including the effect of small interfering RNA on the cell responses. Thapsigargin-induced ER-stress caused only apoptosis after ∼2 hr with Ire1 phosphorylation, and Grp78, ATF4, and CHOP expressions. On the other hand, NaF- and tunicamycin-induced ER-stress caused only autophagy in the early stage by ∼8 hr with ATF4 expression and without CHOP expression. ATF4-siRNA completely inhibited the autophagy induced by NaF or tunicamycin with suppressed ATF4 protein and mRNA expressions, and also inhibited apoptosis by thapsigargin with suppression of both ATF4 and CHOP. CHOP-siRNA had no effect on autophagy activation by NaF and tunicamycin. On the other hand, CHOP-siRNA activated autophagy in thapsigargin-induced ER-stress with significant ATF4 expression, and suppressed apoptosis with CHOP suppression. These results showed that ATF4 is the key signal for autophagy induced by ER-stress, and that autophagy is switched to apoptosis by subsequent CHOP upregulation, suggesting that the changeover switch between autophagy and apoptosis is located between ATF4 to CHOP in the PERK pathway.

## Introduction

Cells have evolved elaborate mechanisms to ensure that proteins are folded and assembled accurately before transport to other organelles. Only correctly folded proteins are allowed to leave the ER. Abnormalities such as unfolded protein accumulation in the ER are collectively called ER-stress (Schröder and Kaufman, 2005; Faitova et al., 2006). ER-stress causes the cell to activate self-protective mechanisms, called the unfolded protein response (UPR), mediated through three ER-stress sensor proteins (Ire1, AtF6, and PERK) located in the ER membrane (Mori, 2000). The UPR initially improves the folding and degradation of unfolded proteins, but if the UPR is overwhelmed, apoptosis could be initiated. Grp78/Bip and GADD153/CHOP are canonically upregulated during apoptosis induced by ER-stress. The Ire1 and PERK pathways cooperate to elicit the maximum response to ER-stress (Harding et al., 2002; Mori, 2003). However, ER-stress induced by unfolded protein accumulation in the ER can also induce autophagy (Ogata et al., 2006; Yorimitsu et al., 2006).

Autophagy is an evolutionarily conserved process involving the formation of double-membraned autophagosomes. These autophagosomes encapsulate the cytoplasmic contents, including the organelles, and deliver this cargo to the lysosome for degradation. Autophagy has emerged as a multifunctional pathway involved in the response to microenvironmental or cellular stress in multicellular organisms (Kuma et al., 2004; Shimizu et al., 2004; Semenza, 2008). Autophagy and apoptosis are important and interconnected stress-response mechanisms. However, the regulatory association of autophagy with UPR is not well understood. Multiple connections must exist between ER-stress, autophagy, and apoptosis. Therefore, the molecular interactions and functional relationships between the signal pathways of these stress-response mechanisms are now receiving considerable attention. In particular, identification of the changeover switch between autophagy and apoptosis in the UPR has become increasingly important.

Our previous study indicated that *in vivo* NaF treatment to rats extensively induced intracisternal granules in the ER lumen and autophagosomes in the cytoplasm of the exocrine pancreas cells, and this autophagy activation was associated with activation of the PERK-eIF2α-CHOP pathway but not with activation of the Ire1-XBP1 pathway (Ito et al., 2009). Other studies have also identified the PERK-eIF2α pathway as required for autophagy (Kouroku et al., 2007; Salazar et al., 2009). In particular, in addition to autophagy activation via PERK-eIF2α-TRB3-Akt/mTor by ER-stress induced by cannabinoid, the autophagy was located on upstream of the apoptosis induced by cannabinoid through ER-stress/UPR (Salazar et al., 2009). Recent studies have indicated that the PERK pathway is important to induce autophagy as a survival pathway in response to several cellular insults (Kim et al., 2010; Rouschop et al., 2010; Rzymski et al., 2010; Avivar-Valderas et al., 2011; Yu et al., 2011). In cellular adaptation to tumor hypoxia, the hypoxia activates autophagy through PERK-dependent expressions of ATF4 and CHOP (Rouschop et al., 2010; Rzymski et al., 2010). The accumulated data strongly suggest that ATF4 is important in the activation of autophagy, but the functional relationship between autophagy, ATF4, and CHOP, and the changeover mechanism between autophagy and apoptosis are not sufficiently understood.

The present study tried to identify the changeover switch between ER-stress induced autophagy and apoptosis by examining the changes in the UPR-related signals and cell-death profile with time in HepG2 cells exposed to three different ER-stress inducers (NaF, tunicamycin (Tu) and thapsigargin (Tg)), including the effect of small interfering RNA on the cell responses to the stress inducers.

## Results

### Effects of three different ER-stress inducers, NaF, Tu, and TG, on cell viability, autophagy activation, and apoptosis induction

**Fig. 1.**
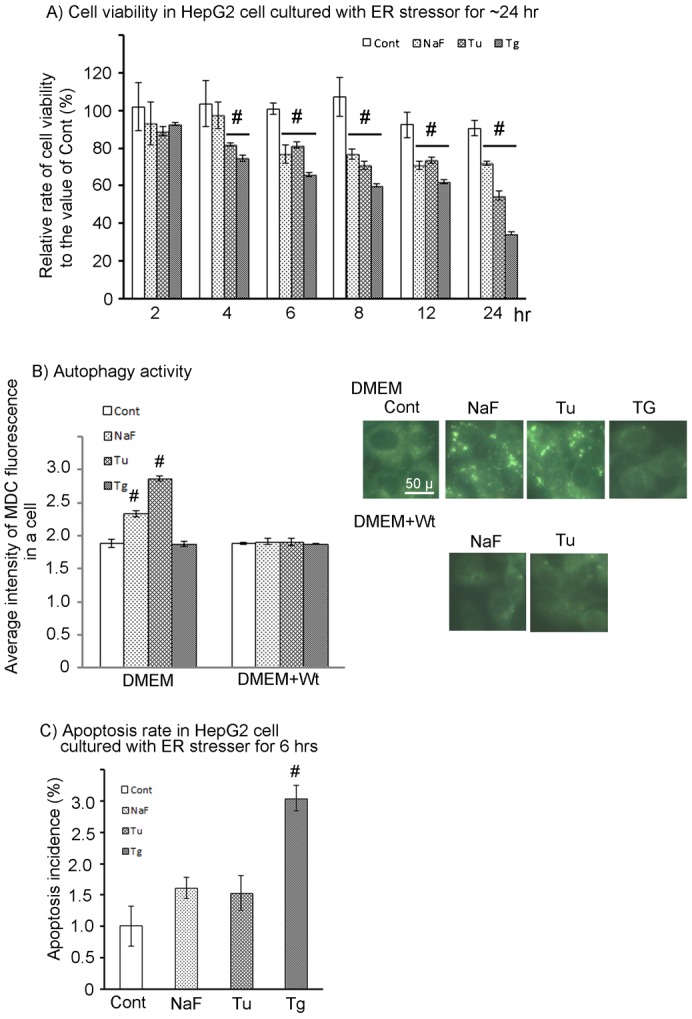
Effects of ER-stress inducers, NaF, tunicamycin (Tu) and thapsigargin (Tg), on cell viability, autophagy, and apoptosis in HepG2 cells. (A) Cell viability in HepG2 Cells exposed to ER-stress inducers for 0∼24 hr was measured with the MTT assay (Cell Counting Kit of Dojindo). (B) Densitometric analysis of autophagy activity in HepG2 cells exposed to ER-stress inducers for 6 hr was performed with MDC. The activity was determined as the fluorescence intensity of MDC incorporated into the cell cultured with each ER-stress inducer in the presence or absence of wortmannin (Wt). Cell image under the fluorescent microscope is shown in the right panel. Lower row photographs of the panel indicate the Wt-treated cells. (C) Apoptosis in HepG2 Cells exposed to ER-stress inducers for 6 hr was examined with Hoechest33342 staining. Apoptosis was identified as the nuclear staining pattern. The incidence of apoptosis is indicated as the percentage of apoptotic cells to total cells in a selected area. Photograph of apoptosis is indicated in the upper panel of Fig. 6. Methodological details of morphometrical and biochemical analysis are given in Materials and Methods. Data are mean ± SD of three individual experiments. #*P*<0.05. Scale bar: 50 µm.

Cell viability at each treatment-culture period was relatively determined compared to the value for 48 hr-precultured cells measured with the MTT assay kit. The viability of the control group at each treatment-culture period remained high at more than 90% in treatment-culture periods from 0 hr to 24 hr (range; 101.9±12.9 to 90.5±3.9%, 100.8±3.0% at 6 hr) (Fig. 1A). The viability in the NaF treatment group was significantly decreased to 76.9±5.0% at 6 hr (Fig. 1A), but then remained stable at more than 70% up to 24 hr (range; 70.7±2.0 to 76.7±2.9%). The viability in the Tu and Tg treatment groups significantly decreased at 4 hr to 81.9±0.6% and 74.6±1.5%, respectively, and to 81.5±1.5 and 66.0±1.2% at 6 hr, respectively (Fig. 1A). The viability in the Tu treatment group remained stable in the range 70.8±2.0 to 81.5±1.5% by 12 hr, but then was severely decreased to 54.7±2.4% at 24 hr. The viability in the Tg treatment group remained stable at 60.2±0.8% to 66.0±1.2% from 6 to 12 hr, and then strongly decreased to 34.7±0.9% at 24 hr. Under these conditions of reduced cell viability, autophagy activity and/or apoptosis incidence were measured at 6 hr and 24 hr during treatment culture.

MDC is incorporated into the autophagosome and emits green fluorescence under ultraviolet excitation. The autophagosomes induced by ER-stress inducers were detected as green fluorescent granules in the cytoplasm, mainly around the nucleus, under fluorescence microscopy (Fig. 1B; NaF and Tu in upper row of right panel). Both NaF and Tu treatments significantly increased fluorescence density in the cytoplasm at 6 hr resulting from the formation of numerous MDC-containing granules (Fig. 1B; left column group of the graph). PI3 kinase inhibition with wortmannin (Wt) completely suppressed the autophagosome formation induced by NaF and Tu treatments (Fig. 1B; right column group of the graph, and NaF and Tu in lower row of right panel). Similarly, NaF and Tu treatments induced many autophagosomes in HepG2 cells at 24 hr, and Wt completely suppressed the autophagosome formation (data not shown). On the other hand, Tg treatment did not activate autophagy at either 6 hr or 24 hr with no relationship to Wt treatment (Fig. 1B).

The effects of ER-stress inducers on apoptosis induction were investigated with Hoechest33342 staining, which detects apoptotic cells with fragmented and chromatin-aggregated nuclei as blue highly fluorescent dots under ultraviolet excitation. Tg treatment caused significant increase in apoptosis at 6 hr (Fig. 1C; 3.0±0.2% for Tg versus 1.0±0.3% for control) and further increase at 24 hr (8.3±0.2%), although the incidence in the control group also slightly increased at 24 hr (1.7±0.2%). On the other hand, neither NaF nor Tu treatment increased apoptosis significantly at 6 hr (Fig. 1C; 1.62±0.2% for NaF and 1.53±0.3% for Tu). However, NaF and Tu treatment slightly, but significantly, increased apoptosis at 24 hr (3.4±0.3% for Tu and 2.7±0.3% for NaF).

### Signal changes on ER-stress sensor pathways with different ER-stress inducers

As described above, autophagy was activated by NaF and Tu treatment, but not by Tg treatment, and apoptosis was not induced in the early phase of treatment with these ER-stress inducers, except for Tg. Signal changes in the ER-stress sensor pathways were investigated by western blotting for phospho-eIF2α, ATF4, CHOP, phosphor-Ire1, Ire1, and GRP.

#### Signal changes in the PERK pathway

**Fig. 2.**
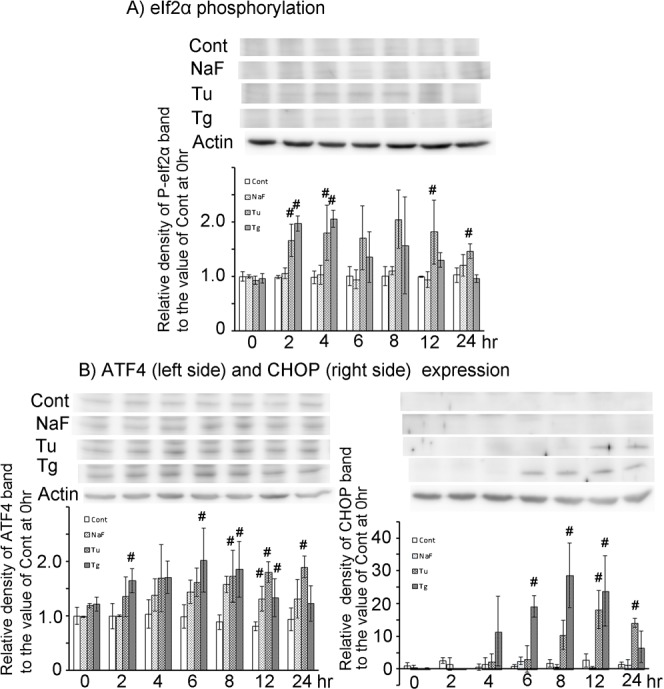
Expression changes in time course of signal proteins on PERK pathway in HepG2 cells exposed to three different ER-stress inducers. Whole cell lysates were prepared for phosphorylated eIF2α, ATF4, and CHOP western blotting from HepG2 cells treated for 0∼24 hr with ER-stress inducers (NaF, Tu and Tg). Protein expression during ER-stress inducer treatment for 0∼24 hr was measured by densitometry of the positive band in each western blot, as described in Materials and Methods. Images are representative of three individual experiments. (A) Time-dependent change of phosphorylated eIF2α level. (B) Change of ATF4 protein expression in the left panel, and change of CHOP in the right panel. Each blot lane time point corresponded to the time point of each column group in lower column graph. Data are mean ± SD of three individual experiments. #*P*<0.05, versus the value of control at 0 hr.

NaF treatment did not increase the phosphorylation level of eIF2α throughout the culture period, whereas ATF4 expression was substantially increased from 4 hr to 24 hr to 1.31∼1.58 times the control value at 0 hr with the peak at 8 hr. Despite this increase in ATF4 expression, CHOP expression was never induced by NaF treatment (Fig. 2). Tu treatment significantly increased the phosphorylation level of eIF2α at 2 hr, and continued to 24 hr. Increased ATF4 expression closely followed the increase in eIF2α phosphorylation with slight lag time, and continued to 24 hr with significant increase at 8–24 hr. CHOP expression was slightly observed at 8 hr and significantly increased during the late phase at 12–24 hr (Fig. 2). Tg treatment significantly increased the phosphorylation level of eIF2α at 2 hr. This increase in phosphorylated eIF2α level was associated with significant increase in ATF4 expression with the same time course and CHOP expression with 4 hr lag time (Fig. 2). eIF2α phosphorylation activity peaked at 4 hr, ATF4 expression at 6 hr, and CHOP expression at 8 hr. These activated levels gradually decreased to the baseline level toward the end of treatment culture (24 hr).

#### Signal changes in the Ire1 pathway

**Fig. 3.**
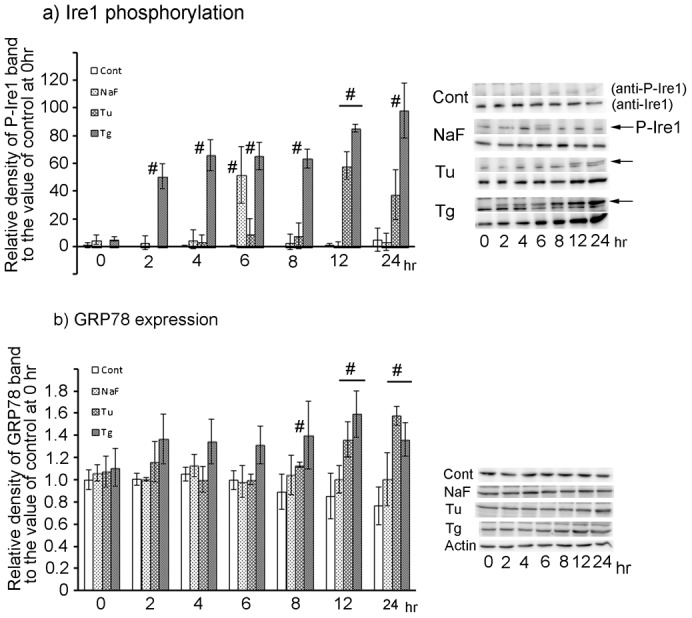
Expression changes with time of signal proteins on the Ire1 pathway in HepG2 cells exposed to three ER-stress inducers. Whole cell lysates were prepared for Ire1, phosphorylated Ire1, and GRP78 western blotting from HepG2 cells treated for 0∼24 hr with ER-stress inducers (NaF, Tu, and Tg). Patterns of protein expression for Ire1, phosphorylated Ire1, and GRP78 during ER-stress inducer treatment for 0∼24 hr was examined with western blotting, as described in Materials and Methods. Images are representative of three individual experiments. (A) Time-dependent change of phosphorylated Ire1 level in upper position of each treatment group, and change of Ire1 protein expression in lower position in each. Arrow indicates phosphorylated Ire1 band in each ER-stress inducer. (B) Time-dependent change of GRP78 expression is indicated. Quantification of protein expression was performed with densitometry of the positive band in each western blot. Data are mean ± SD of three individual experiments. #*P*<0.05, versus the value of control at 0 hr. Data are mean ± SD of three individual experiments. #*P*<0.05, versus the value of control at 0 hr.

NaF treatment induced significant transient increase of Ire1 phosphorylation at 6 hr, but caused no change in GRP 78 expression throughout the treatment culture (Fig. 3). Tu treatment significantly increased Ire1 phosphorylation and protein expression after 12 hr, and also increased GRP78 expression with the same time pattern. Tg treatment significantly increased Ire1 phosphorylation and protein expression after 2 hr, and also significantly increased GRP78 expression after 8 hr with the peak at 12 hr. These results combined with the findings for autophagy or apoptosis induction indicate that autophagy required ATF4 expression but not CHOP expression, and that apoptosis was induced with increased expression of ATF4, CHOP, and GRP-78. To investigate the involvement of ATF4 and CHOP in the cell protective response, the effects of their knockdown on autophagy activation and apoptosis induction were examined using siRNA.

### Effects of ATF4 and CHOP siRNAs on autophagy activation and apoptosis induction by ER-stress inducers

#### Effects of ATF4 and CHOP siRNAs on expression of mRNA and protein

**Fig. 4.**
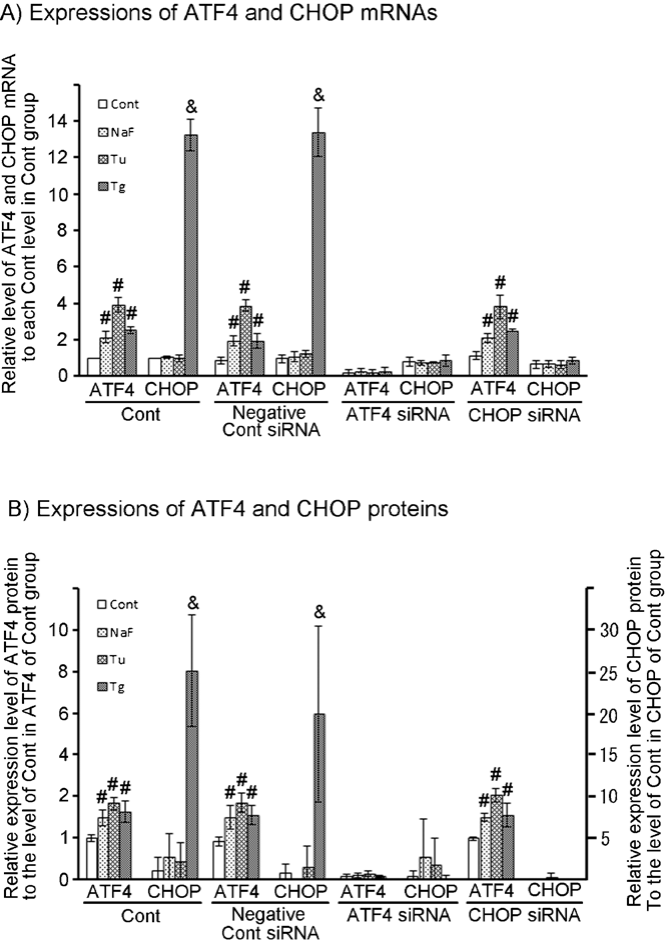
Effects of ATF4 and CHOP siRNA on expression of mRNAs and proteins in HepG2 cells exposed to three ER-stress inducers. Small interfering RNA (siRNA) transfections with a non-targeting siRNA (negative control siRNA) or ATF4 or CHOP siRNA were performed in HepG2 cells, according to the manufacturer's instructions. After the first 48 hr pre-incubation in DMED containing siRNA, each ER-stress inducer is added to the medium and the culture continued for 6 hr to induce ER stress. (A) Relative mRNA levels of ATF4 and CHOP in ATF4 or CHOP siRNA knockdown, negative control siRNA addition (non-targeting siRNA addition) and control (control; no-addition of siRNA) HepG2 cells were measured by quantitative real-time PCR (qPCR). The qPCRs were performed in duplicate for each sample. (B) Protein levels of ATF4 and CHOP in ATF4 or CHOP siRNA knockdown, negative control siRNA addition and control HepG2 cells were measured by densitometry for western blotting. Samples were collected from three individual experiments. Data are mean ± SD of three individual experiments. #,&*P*<0.05, versus the value of each control.

The effects of the ATF4 siRNA and CHOP siRNA on expression of relative mRNAs and proteins were investigated with quantitative real-time RT-PCR and western blotting. Expression of both mRNA and protein of ATF4 were significantly elevated by NaF, Tu, or Tg treatments for 6 hr (Fig. 4A,B). Expression of both mRNA and protein of CHOP were not induced by NaF and Tu treatments for 6 hr, but were highly induced by Tg treatment for 6 hr (Fig. 4A,B). The same expression of mRNAs and proteins of ATF4 and CHOP were found in the presence of negative control siRNA.

Addition of ATF4 siRNA completely suppressed the expression of mRNA and protein of ATF4 by all three ER-stress inducers, resulting in suppression of CHOP which is located downstream of ATF4 (Fig. 4A,B). Addition of CHOP siRNA completely suppressed the expression of mRNA and protein of CHOP, but had no effect on expression of those of ATF4 (Fig. 4A,B).

#### Effects of ATF4 and CHOP siRNAs on autophagy activation and apoptosis induction

**Fig. 5.**
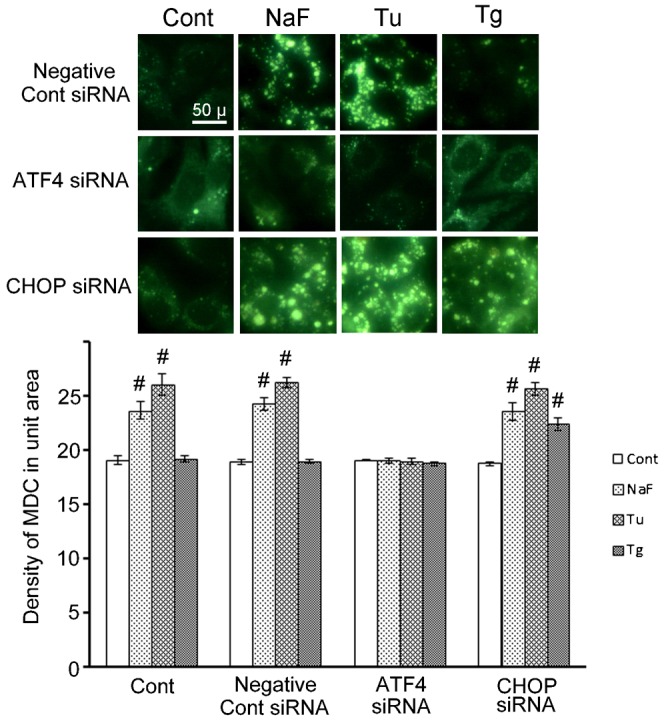
Effects of ATF4 or CHOP siRNA on autophagy activation in HepG2 cells treated with ER-stress inducers. siRNA transfections with a non-targeting siRNA (negative control siRNA) or ATF4 or CHOP siRNA were performed, as described in the legend of Fig. 4. Densitometric analysis of autophagy activity in HepG2 cells exposed to ER-stress inducer treatments was conducted with MDC. The activity was determined with fluorescence intensity of MDC incorporated into the treated cells. Upper row photographs of upper panel show fluorescence microphotographs of the cells cultured with negative control siRNA in the presence of each ER-stress inducer. Middle row photographs show cells exposed to ATF4 siRNA. Lowest row photographs are cells exposed to CHPO siRNA. Data are mean ± SD of three individual experiments. #*P*<0.05, versus the value of each control. Scale bar: 50 µm.

**Fig. 6.**
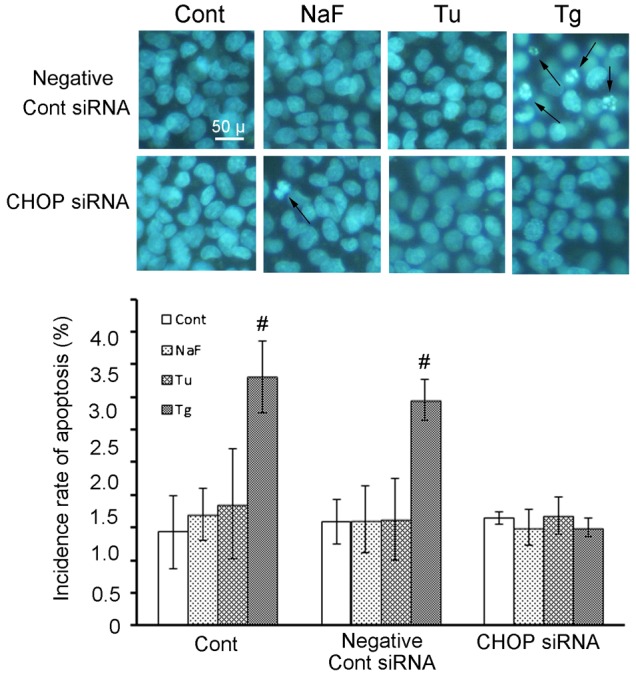
Effect of CHOP siRNA on apoptosis induction in HepG2 cells treated with ER-stress inducers. siRNA transfections with a non-targeting siRNA (negative control siRNA) or CHOP siRNA were performed, as described in the legend of Fig. 4. Apoptosis was identified with nuclear staining pattern of Hoechest33342. Upper row photographs of upper panel show fluorescence microphotographs of the cells cultured with negative control siRNA in the presence of each ER-stress inducer. Lower row photographs show cells exposed to CHOP siRNA and three different ER-stress inducers. The incidence of apoptosis is indicated as the percentage of apoptotic cells to total cells in a selected area. Data are mean ± SD of three individual experiments. #*P*<0.05, versus the value of each control. Scale bar: 50 µm.

Addition of negative control siRNA did not have any effect on autophagy activation and apoptosis induction induced in HepG2 cells by ER-stress inducers: that is, NaF- or Tu-treated cells exhibited elevated autophagy activity, but not apoptosis, and Tg-treated cell exhibited apoptosis, but not autophagy (Fig. 5 for autophagy and Fig. 6 for apoptosis).

Addition of ATF4 siRNA to NaF- or Tu-treated cells completely suppressed the autophagy that was activated by these ER-stress inducers (Fig. 5, middle row of upper panel and lower panel), associated with suppression of both ATF4 and CHOP (Fig. 4A,B).

Addition of CHOP siRNA to NaF- or Tu-treated cells did not suppress the autophagy induced by these ER-stress inducers (Fig. 5, lowest row of upper panel and lower panel graph). On the other hand, addition of CHOP siRNA to Tg-treated cells inhibited the apoptosis induced by this ER-stress inducer (Fig. 6; Tg-CHOP siRNA of lower row in upper panel), associated with suppression of CHOP (Fig. 4B). Interestingly, addition of CHOP siRNA to Tg-treated cells activated autophagy with increased ATF4 expression, but with suppression of CHOP expression (Fig. 5; Tg-CHOP siRNA of lowest row in upper panel and lower panel photograph).

## Discussion

Excessive ER-stress caused by high loads of unfolded protein in the ER is well known to induce caspase-mediated apoptosis through the UPR. Autophagy is also linked to the ER-stress/UPR pathways (Yorimitsu and Klionsky, 2007; Hoyer-Hansen and Jäättelä, 2007). However, little is known about whether the process of autophagy regulates the UPR pathway and how specific UPR targets might control autophagy. The present study of the change-over mechanism between autophagy and apoptosis induced by the ER-stress/UPR pathway found that autophagy activated by NaF and Tu treatments was associated with increased ATF4 expression, but not with CHOP expression, at ∼8 hr after treatment, and that addition of ATF4 siRNA to the NaF and Tu treatment cultures inhibited such autophagy activation with suppression and inhibition of ATF4 and CHOP expression, whereas CHOP siRNA treatment had no effects on autophagy induction with maintained elevated ATF4 level. In contrast, apoptosis induced by Tg treatment was completely suppressed by addition of CHOP siRNA to the Tg treatment culture, but instead induced autophagy with increased ATF4 and suppressed CHOP expression. Based on these findings, we concluded that ATF4 expression in the PERK pathway is the key signal for autophagy activation by ER-stress, and that autophagy is switched to apoptosis by subsequent upregulation of CHOP expression.

ER-stress induced by the accumulation and aggregation of unfolded proteins activates the UPR, a cellular adaptive response that leads to inhibition of protein translation through the co-ordinate action of ER-stress sensors (Mori, 2000; Ron and Walter, 2007). Phosphorylation of eIF2α by PERK reduces global protein synthesis, but results in preferential translation of selected mRNAs including ATF4 (Harding et al., 1999; Koumenis et al., 2002). ATF4 is a mediator of the integrated stress response, a gene expression program involved in oxidative stress, amino acid synthesis, differentiation, and metastasis angiogenesis (Harding et al., 2003; Blais et al., 2004). ATF4 is well known to act as a protective factor during cellular stress (Ameri and Harris, 2008), and the pro-death function of ATF4 has been attributed to its regulation of CHOP (Zinszner et al., 1998; Armstrong et al., 2010; Hill et al., 2009). Although ER-stress induced by tunicamycin caused renal cellular dysfunction and ultra-structural changes in both *chop*+/+ and *chop*−/− mice, apoptosis caused by tunicamycin significantly decreased in the cells of *chop*−/− mice, suggesting that CHOP is involved in the induction of apoptosis in response to ER-stress (Zinszner et al., 1998), and CHOP is indispensable for cell death in response to ER-stress (Armstrong et al., 2010).

On the other hand, ER-stress is also a potent inducer of autophagy (Yorimitsu et al., 2006; Yorimitsu and Klionsky, 2007). The PERK/eIF2α pathway of the UPR has been implicated in autophagy regulation (Kouroku et al., 2007; Tallóczy et al., 2002). In addition, MAP1LCB3 and ATG5 are both central in the ubiquitin-like conjugation systems involved in the formation of autophagosomes (Yorimitsu and Klionsky, 2005). Recent studies of regulation of autophagosome formation have provided the direct link implicating the PERK-dependent transcription factors ATF4 and CHOP in the translational activation of *MAP1LC3B* and *ATG5* during hypoxia (Rouschop et al., 2010; Rzymski et al., 2010). Knockdown of ATF4 prevented *MAP1LC3B* induction during hypoxia (ER-stress), but *MAP1LC3B* induction was not prevented by CHOP knockdown, and CHOP knockdown lead to a reduction in ATG5 (Rouschop et al., 2010). Additionally, ATF4-mediated induction of MAP1LCB3 was suggested to function to replenish MAP1LCB3 level during stress conditions characterized by high autophagic flux, but is not required for activation of autophagy. These previous studies identified the PERK/eIF2α pathway as necessary for autophagy.

In the present study, transient knockdown of ATF4 with siRNA also inhibited the autophagosome formation induced by different ER-stress inducers. In agreement with the previous studies, the present findings for ATF4 expression identified the ATF4 signal on the PERK pathway as necessary for autophagy. However, the response profile induced by CHOP in this study was different from those of the previous two studies. The present study showed that CHOP expressed by Tg and Tu (at and after 8 hrs of treatment) was involved in apoptosis occurrence, and that CHOP siRNA treatment completely suppressed apoptosis, and instead activated autophagy with increased ATF4 and suppressed CHOP expression. These present findings indicate that the change-over switch between autophagy and apoptosis is located between the ATF4 to CHOP of the PRRK signal pathway. On the basis of these findings including the present results (Figs 5, 6), we conclude that ATF4 is the key signal for autophagy induced by ER-stress, and that the selector switch between autophagy and apoptosis is located within the process that preferentially transmits ATF4 expression to CHOP expression.

ATF4-mediated MAP1LCB3 may be required for maintaining continuous autophagy, but not for activation of autophagy (Rouschop et al., 2010). The downstream signals of ATF4 that participate in direct induction of ER-stress-dependent autophagy and in selective inhibition of its downstream CHOP remain unclear. The essential autophagy gene *ULK1* is transcriptionally upregulated by direct activity of ATF4 at the gene *ULK1* promoter, suggesting ATF4 is important in the initiation of autophagy (Pike et al., 2013). ER-stress induced in HepG2 cells by NaF or Tu treatments gradually suppressed the phosphorylation of Ulk1 [Ser757], target molecule of mTor, at 2–4 hr after the treatments, but the protein expression of Ulk1 in the treated HepG2 cells was increased with NaF during 2–24 hr after the treatment and with Tu during 2–12 hr (T.M., S.M., H.N., K.N. and S.M., unpublished data). It is likely that more studies are required to clarify interpretation of Ulk1 in ER-stress inducing autophagy. ATF4 is also known to directly bind and activate an ATF site upstream of the GADD34 promoter (Ma and Hendershot, 2003). Overexpression of the active form of GADD34 that constitutively dephosphorylates eIF2α can attenuate the phosphorylation of eIF2α and severely inhibit the induction of ATF4, suggesting that GADD34 is important as a negative feedback regulator of the PERK signal pathway (Blais et al., 2004). In the present study, Tu treatment induced continuous high levels of eIF2α phosphorylation with decreasing viability related to CHOP expression with time. Sustained eIF2α phosphorylation is apparently lethal in ER-stress exposed cells. We speculate that the connection between autophagy and suppressive CHOP expression is related to the ability of GADD34 to suppress sustained ATF4 expression, but this hypothesis requires further research.

## Materials and Methods

### Antibodies and reagents

The following antibodies and reagents were used: mouse monoclonal antibody specific for β-actin (Sigma–Aldrich, St Louis, MO); rabbit antibodies to ATF4, CHOP (Santa Cruz Biotechnology, Santa Cruz, CA), GRP78 (Sigma–Aldrich), Ire1 (Cell Signaling Technology, Danvers, MA) and Phospho-Ire1 (Novus Biologicals, Littleton, CO) as primary antibodies for western blot analysis; and horseradish peroxidase conjugated goat antibodies to rabbit IgG and mouse IgG (Cappel, Aurora, OH) as secondary antibodies. Hoechest33342 (CALBIOCEM, Darmstadt, Germany) and Monodansylcadaverine (MDC) (Sigma–Aldrich) were used for histochemistry. Thapsigargin and tunicamycin were purchased from Sigma–Aldrich, and NaF from Nakarai TECK (Kyoto, JAPAN). HepG2 cell (Human Hepatocellular Carcinoma Cell Line) was portioned from Health Science Research Resource Bank (Sen-nan City, JAPAN).

### Cell culture, ER-stress induction, and assessment of autophagy, apoptosis, and cell viability

HepG2 cells were maintained in Dulbecco's modified Eagle medium (DMEM, Nissui Pharmaceutical, Tokyo, Japan) containing 10% fetal bovine serum (FBS, Nichirei Biosciences, Osaka Japan) at 37°C. After pre-culture for 48 hr at 1.0×10^5^ cells/ml cell density, the cells were treated with NaF (1 M in phosphate buffered saline) at 1 mM final concentration, tunicamycin (2 mg/ml in 1 N NaOH) at 4 µg/ml, and thapsigargin (2 mM in DMSO) at 250 nM for the indicated times to induce ER-stress. To inhibit autophagy in all treatment groups, PI3 kinase inhibitor wortmannin (Wt) was added to the culture medium in each treatment group at 300 nM final concentration.

To assess autophagy activity, the treated cells were stained at 100 µM final concentration with MDC (100 mM in the solution of acetic acid/DMEM = 1:5) at 37°C for 45 min (Biederbick et al., 1995). HepG2 cells were observed by fluorescent microscopy (IMT-2) with a source using 335 nm excitation and 512 nm emission. For morphometrical analysis for autophagy activity, 5 areas in a 6-cm culture dish were selected at random and images taken at 400 times magnification under the microscope. Then, 5 cells in each area were randomly selected and fluorescence intensity was measured in each cell with Scion image. The mean value of the fluorescence intensity measured in 25 cells was evaluated as autophagy activity in a sample. Three individual experiments were repeated.

To assess apoptosis, the treated cells were stained at 2 µg/ml final concentration with Hoechest33342 (1 mg/ml in distilled water) at 37°C for 30 min (Yao et al., 2011). The cells were observed by fluorescent microscopy with a source using 346 nm excitation and 460 nm emission. For morphometrical analysis for apoptosis incidence, 5 areas in a 6-cm culture dish were randomly selected and images taken at 200 times magnification under the microscope. Numbers of living cells containing clear round nucleus and apoptosis cells containing fragmented nucleus within the selected 5 areas were counted in each treatment group. Apoptosis incidence was evaluated as the mean values of these findings. Three individual experiments were repeated.

Cell viability was assessed with the Cell Counting Kit (Dojindo, Kumamoto, Japan) according to manufacturer's instructions. The cell viability (absorbance value: Abs) at each point in each treatment group was measured at 450 nm with a mulch-plate reader (2030ARVO™X, PerkinElmer, Kanagawa Japan) and was indicated as a relative value to the viability of the no-treatment group at 0 hr after 48 hr pre-culture [Calculation formula: Viability (%) = (Sample Abs−blank Abs)/(control at 0 hr point Abs−blank Abs)]. Three individual experiments were repeated.

### Western blot analysis

The cells treated with ER-stress inducers were lysed with 1% Triton HEPES buffer, pH 7.5, (20 mM HEPES, 150 mM NaCl, 1% Triton X-100, 10% glycerol, 1 mM EDTA-2Na, 10 mM sodium pyrophosphate, 100 mM NaF, 17.5 mM glycero-2-phosphate disodium salt hydrate, 1 m M PMSF, 4 mg/ml aprotinin and 2 µg/ml pepstatin). Whole cell lysates were resolved by SDS/PAGE (in 7.5%, 10% or 12% SDS polyacrylamide gel), electro-blotted onto Immobilon-p Transfer Membrane (Merck Millipore, Darmstadt Germany), and probed with the indicated antibodies (ATF4 = 1:200; CHOP = 1:500; Phospho-eIF2α = 1:1000; GRP78 = 1:1000; Ire1 = 1:1000; Phospho-Ire1 = 1:1000; β-actin = 1:20,000). Horseradish peroxidase conjugated goat antibodies to rabbit IgG (1:1000) and mouse IgG (1:1000, for mouse monoclonal antibody specific for β-actin) were used as second antibodies. After incubation of the second antibody for 1 hr at room temperature, the corresponding bands were detected using ECL (Millipore Co., Billerica, MA) and LAS-3000 (FUJIFUILM Tokyo, Japan).

### siRNA treatment of HepG2 cells

RNA interference-mediated gene knockdown was achieved using pre-validated Quiagen HP small interfering RNAs (siRNAs) for *ATF4* (SI03019345) and *CHOP* (SI00059528). All siRNA experiments incorporated a validated negative control siRNA (Quiagen AllStars negative control siRNA). siRNA knockdown experiments were carried out by placing 0.6×10^5^ cells (dispersed in 500 µl DMEM containing 10% FCS) in 24-well plates with a mixture of 6 pmol siRNA (0.3 µl), 100 µl Opti-MEM I Reduced-Serum Medium (Invitrogen), and 1 µl Lipofectamine RNAiMAX Reagen (Invitrogen) that was pre-incubated for 20 min according to the manufacturer's instructions. Cells were then cultured for 48 hr prior to drug treatment for 6 hr. Cultures after treatment were processed as described above.

### Quantitative real-time RT-PCR analysis

Total RNA was extracted from HepG2 cells using the GenElute Mammalian Total RNA Miniprep Kit (Sigma–Aldrich Co., St Louis, MO) according to the manufacturer's instructions. Expression levels of human ATF4, human CHOP, and housekeeping GAPDH (rat) mRNA were determined using the specific primer indicated in Table 1. The Power SYBR Green RNA-ti-CtTM 1-step Kit (Life Technology Co., Carlsbad, CA) was used to detect the quantitative real-time PCR products according to the manufacturer's instructions. The incubation conditions were as follows: cDNA synthesis at 48°C for 30 min, predenature at 95°C for 10 min, followed by 40 cycles of 15 sec at 95°C, annealing for 60 sec at 60°C, and extension for 60 sec at 60°C. PCRs for each sample were done in triplicate for both the target genes and the GAPDH.

**Table 1.**
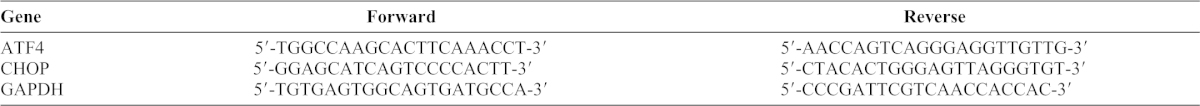
Quantitative PCR primers.

### Statistical analysis

Results are presented as the mean ± SD of the indicated number of separate individual experiments, each performed at least in triplicate. After confirming equal variances by Bartlett's test, Tukey's multiple comparison was used to compare morphometrical and densitometric measurements.
